# Effect of Crystal Orientation on Dislocation Loop Evolution Under Electron Radiation in Pure Aluminum

**DOI:** 10.3390/ma19020350

**Published:** 2026-01-15

**Authors:** Yupeng Yin, Qianfei Feng, Wentuo Han, Xiaoou Yi, Pingping Liu, Kenta Yoshida, Koji Inoue, Qian Zhan, Somei Ohnuki, Farong Wan

**Affiliations:** 1 School of Materials Science and Engineering, University of Science and Technology Beijing, Beijing 100083, China; yinyupengustb@163.com (Y.Y.); b20200659@xs.ustb.edu.cn (Q.F.); xiaoouyi@ustb.edu.cn (X.Y.); ppliu@ustb.edu.cn (P.L.); qzhan@mater.ustb.edu.cn (Q.Z.); ohnuki@air.ocn.ne.jp (S.O.); wanfr@mater.ustb.edu.cn (F.W.); 2 State Key Laboratory of Nuclear Power Safety Technology and Equipment, University of Science and Technology Beijing, Beijing 100083, China; 3 Oarai Center, Institute of Material Research, Tohoku University, Oarai 311-1313, Ibaraki, Japan; k_yoshida@imr.tohoku.ac.jp (K.Y.); koji.inoue.e2@tohoku.ac.jp (K.I.); 4 School of Engineering, Hokkaido University, Sapporo 060-8628, Hokkaido, Japan

**Keywords:** electron irradiation, crystallographic orientation, dislocation loops, self-interstitial atoms, threshold displacement energy

## Abstract

Aluminum, the primary structural material used in spacecraft, operates in low Earth orbit (LEO). It is subjected to high-energy electron irradiation with energies ranging from 0.1 to 10 MeV, which produces significant irradiation damage. Understanding the characteristics of irradiation defects with crystallographic orientations is crucial for analyzing the failure of spacecraft components and for developing aerospace materials with improved irradiation resistance. In this study, pure aluminum was irradiated in situ at room temperature using 200 kV transmission electron microscopy. The irradiation defects were comparatively analyzed for four crystallographic orientations, focusing on the size, density, and interstitial content of <111> and <110> dislocation loops. For all four irradiation directions, the interstitial atom density (IAD) within <111> loops is significantly higher than that in <110> loops. Notably, under [110]-direction irradiation, IAD in <111> loops is approximately 55 times that in <110> loops. This phenomenon is attributed to the one-dimensional migration of <110> loops. Among the four irradiation directions, the total IAD in the two types of loops decreases in the order: [110] > [111] > [310] > [100]. The threshold displacement energy (E_d_) of aluminum at room temperature is inferred to follow the relationship: [110] < [111] < [310] < [100].

## 1. Introduction

Aluminum and its alloys are widely utilized in the aerospace industry [1,2]. For example, AA6061 aluminum alloy is extensively employed in structural components such as satellite primary structures, solar array support frames, and ring-frame structural modules of space stations [3]. In contrast, AA2219 aluminum alloy is predominantly used in rocket propellant tanks and large sealed compartments of manned spacecraft [4]. Satellites, space stations, and other spacecraft operating in low Earth orbit (LEO) experience harsh service environments, where external surface temperatures fluctuate between 93 and 453 K [5], and continuous exposure to radiation from high-energy charged particles such as electrons and protons occurs [6,7,8].

To date, research on aluminum irradiated by space particles has been relatively limited. However, extensive studies have been conducted on the mechanical properties of aluminum alloys. AA6061-T6 aluminum alloy exhibits excellent mechanical performance at low temperatures. At 77 K, the yield strength, ultimate tensile strength, and elongation to failure increase by approximately 18%, 33%, and 53%, respectively. After cyclic stress loading at 108 K, the alloy shows pronounced cyclic hardening behavior, accompanied by an approximately 143% improvement in fatigue life [9]. Previous studies have demonstrated that low-temperature deformation leads to pronounced dislocation tangling near grain boundaries, whereas at room temperature, dislocations tend to pass through grain boundaries. Compared with room temperature, the altered dislocation behavior in aluminum alloys under low-temperature conditions results in different mechanical properties [10].

In contrast, studies on the effects of high-energy particle irradiation on the mechanical properties of aluminum alloys have predominantly focused on neutron irradiation. Neutron-induced nuclear transmutation reactions generate gaseous elements such as hydrogen and helium, which are among the key driving factors for irradiation swelling. Meanwhile, neutron irradiation introduces defects, such as dislocations and voids, in aluminum alloys and is accompanied by the formation of transmutation-induced silicon. The combined effect of these factors leads to irradiation hardening and embrittlement, thereby significantly altering the mechanical properties of aluminum alloys [11,12,13]. For example, earlier neutron irradiation studies on 6061 aluminum alloy demonstrated that high-dose irradiation leads to significant irradiation hardening and a corresponding decrease in ductility [12]. Studies on neutron irradiation effects in cold-worked commercial A5 aluminum have shown that irradiation induces silicon precipitation due to transmutation reactions, while the dislocation density introduced by cold working decreases with increasing neutron dose, accompanied by enhanced strength and ductility [14].

Neutron irradiation studies are mainly motivated by applications in nuclear reactor environments, whereas the dominant irradiation particles in space environments are electrons and protons [6,7,8]. Electron irradiation fundamentally differs from neutron irradiation in terms of energy transfer mechanisms and defect evolution processes [15]. Neutrons, especially fast neutrons, can induce nuclear transmutation reactions in lattice atoms, producing complex defect structures and compositional changes, which complicate the investigation of irradiation damage mechanisms in aluminum alloys. In contrast, when aluminum and its alloys are irradiated by electrons with energies above a certain threshold, only isolated Frenkel defect pairs are generated in the material, without the formation of displacement cascades [16]. Therefore, electron irradiation provides a more suitable approach for investigating irradiation-induced microstructural defect evolution. However, studies on electron irradiation effects in aluminum and its alloys remain limited, particularly with regard to the influence of electron irradiation direction on the evolution of defects such as dislocation loops.

The radiation environment in near-Earth space is primarily dominated by the inner radiation belt and the lower portion of the outer radiation belt [7,8,17,18]. The inner radiation belt is located at an altitude of approximately 600–10,000 km above the equatorial plane and is mainly composed of high-energy protons and electrons. Proton energies may exceed 100 MeV, while electron energies typically range from approximately 0.1 to 10 MeV. The outer radiation belt extends from about 10,000–60,000 km, and its lower region (approximately 13,500–20,000 km) overlaps with the upper boundary of near-Earth space. This region is predominantly characterized by trapped high-energy electrons with energies up to 7 MeV, accompanied by a small fraction of low-energy protons [17,19,20,21,22]. When the kinetic energy transferred from energetic electrons or protons to a lattice atom exceeds the displacement threshold energy (E_d_), the atom may be displaced from its lattice site, leading to the formation of a Frenkel defect pair consisting of a vacancy and a self-interstitial atom (SIA), which marks the onset of irradiation damage. The irradiation damage behavior of aluminum and its alloys is closely related to the service reliability of spacecraft components and serves as an important basis for failure analysis. Metals such as Al [23], Be [24], and Mg [25,26] exhibit relatively low E_d_. For example, the E_d_ of Al is approximately 16 eV [27,28], which corresponds to a minimum electron threshold energy (E_t_) of about 169 eV required to initiate atomic displacement. Be has an E_d_ of 15 eV with a corresponding E_t_ of 58 eV, whereas Mg exhibits an even lower E_d_ of 10 eV and an E_t_ of 101 eV [29,30]. These values clearly indicate that high-energy electrons present in the LEO environment can readily induce irradiation damage in these metals.

Pure aluminum exhibits a simple face-centered cubic (FCC) crystal structure and contains no alloying elements, second-phase particles, or precipitates, thereby effectively avoiding the additional complexity introduced by solute atoms, precipitate interfaces, and compositional heterogeneity. These factors are known to significantly influence the nucleation, migration, and interaction of irradiation-induced defects in alloy systems, increasing the difficulty of mechanistic interpretation [15]. Moreover, previous studies have shown that, during transmission electron microscopy (TEM) observations of aluminum alloys, precipitates may interfere with the identification and analysis of dislocation loops formed under electron irradiation [31]. Thus, pure aluminum provides a well-defined and simplified material choice for investigating the evolution of irradiation-induced defects, such as dislocation loops, under electron irradiation. More importantly, the insights obtained from pure aluminum provide an essential foundation for understanding irradiation behavior in aluminum alloys. At present, the studies on aluminum alloys under space-relevant particle irradiation, particularly electron irradiation, remain limited. Therefore, elucidating the orientation dependence of irradiation-induced damage in pure aluminum establishes a foundation for subsequent investigations of irradiation damage in aluminum-based structural materials. Addressing this key issue, together with future measurements and analyses of E_d_ in engineering aluminum alloys containing alloying elements such as Si, Mg, and Cu, will further advance the understanding of electron irradiation damage mechanisms in structural materials.

The relatively low E_d_ and E_t_ values of Al allow a 200 keV electron beam in a conventional TEM to induce irradiation damage within the specimen volume [30]. Meanwhile, the nucleation and evolution of irradiation-induced defects can be directly observed and analyzed during electron irradiation, thereby enabling in situ irradiation damage studies. In this context, for irradiation damage research in metals, a standard 200 kV TEM plays a role in low-threshold-energy materials such as Al, Be, and Mg, comparable to that of an ultra-high-voltage electron microscope (UHVEM) on metals with high-threshold energies such as Fe, Cu, and W. The ability to perform in situ irradiation experiments in conventional TEMs, therefore, partially mitigates the limitations caused by the global scarcity of UHVEMs, thereby enabling broader investigations into the fundamental mechanisms of irradiation damage [16].

Li et al. [29] performed in situ observations and analyses to investigate the migration behavior of interstitial loops in hydrogen-implanted pure aluminum during electron irradiation. They reported, for the first time, the long-range one-dimensional (1D) migration of dislocation loops, with migration distances of up to approximately 1500 nm. A well-designed experimental strategy was implemented in which the electron beam irradiation position was deliberately shifted to modify the spatial concentration gradient of irradiation-induced SIAs. Consequently, the migrating dislocation loops were observed to reverse their migration direction. These results demonstrated that the concentration gradient of irradiation-produced SIAs acted as the driving force governing the 1D migration of dislocation loops. Du et al. [32] employed a conventional TEM to perform in situ observations on the evolution of gas bubbles in pure Al implanted with He, Ne, and Ar ions under electron irradiation. Their results showed that all three types of bubbles underwent coalescence and growth when exposed to a 200 keV electron beam. Accompanying the bubble evolution, the selected-area electron diffraction (SAED) patterns revealed a transition in the surrounding matrix from a single-crystal spot pattern to a polycrystalline ring pattern. The authors proposed that under electron irradiation, the high pressure gas inside the bubbles could be ionized into plasma, releasing substantial energy that induces local recrystallization, thereby leading to the formation of polycrystalline structures. Similarly, Li et al. [33] reported bubble growth and rupture in D- and H-ion-implanted Al samples. Consistent with the evolution of bubbles, polycrystalline diffraction rings appeared in the SAED. Furthermore, Chen et al. [34] observed abnormal exothermic behavior in bubbles formed by implantation of D, H, He, Ne, and Ar ions when irradiated with a 200 kV TEM. They proposed that this abnormal heat release is influenced by multiple factors, including bubble morphology and internal pressure, gas species and density, electron beam energy, and irradiation duration.

Although significant progress has been made in in situ irradiation damage studies of Al, the influence of crystallographic orientation on the evolution of dislocation loops in Al has rarely been reported. In this paper, we investigate the effect of crystallographic orientation on the evolution of dislocation loops in aluminum.

## 2. Materials and Methods

The materials used in this study were high-purity aluminum (99.99 wt.%) supplied by Beijing Cuibolin Nonferrous Metals Technology Development Center Co., Ltd., Beijing, China. The as-cast aluminum specimens were machined into 3 mm discs, sealed in evacuated quartz tubes, and annealed at 200 °C for 100 min to remove residual stresses and pre-existing dislocations. The foils were prepared by twin-jet electropolishing to obtain electron-transparent areas with a thickness of approximately 100 nm suitable for TEM observation. The electrolyte consisted of 25% nitric acid and 75% methanol (by volume). Electropolishing was performed at −25 °C with an applied voltage of approximately 20 V using a TenuPol-5 twin-jet electropolisher (Struers, Ballerup, Denmark).

The electron irradiation and in situ observation were performed by a conventional FEI Tecnai F20 TEM (Thermo Fisher Scientific, Hillsboro, OR, USA) with an accelerating voltage of 200 kV. To avoid the influence of grain boundaries on the evolution of irradiation-induced defects, grains with diameters larger than 4 µm were selected during the electron irradiation experiments, and the irradiation area was confined to a circular region with a diameter of 1.2 µm.

In irradiation damage processes, the projection of target atoms onto a plane perpendicular to the incident beam direction plays a particularly important role. The atomic density on this projected plane strongly influences the scattering cross section for irradiation damage as well as the collision probability between incident particles and target atoms. In in situ TEM electron irradiation experiments, the crystallographic orientation of the irradiated crystal and the irradiation direction can be determined using selected-area electron diffraction. The zone-axis direction identified in the electron diffraction pattern corresponds to the irradiation direction of the electron beam. Further details can be found elsewhere [24,25,35,36,37,38]. The electron beam was aligned along four crystallographic orientations: [100], [310], [110], and [111]. For each irradiation condition, the TEM magnification and irradiation area size were kept constant, and the irradiation duration was set to 10 min. After irradiation, two ***g*** vectors were selected for each irradiation direction of the electron beam, and the dislocation loops were analyzed using the two-beam diffraction condition. The dislocation loop size, number density, and spatial distribution formed under electron irradiation along the four crystallographic orientations were statistically evaluated. Based on the extinction rules of dislocation loops with Burgers vectors of $\overset{⃑}{b}\;=\;\frac{1}{3}\;<111>$ (referred to as <111>) and $\overset{⃑}{b}\;=\;\frac{1}{2}\;<110>$ (referred to as <110>) under different ***g*** conditions, the fraction, average size, and number density of both loop types were quantitatively determined. Furthermore, the interstitial atom density (IAD) corresponding to the <111> and <110> loops under different irradiation directions was calculated using the atom-occupied area per unit cell on the relevant crystallographic planes of the FCC lattice.

## 3. Results

### 3.1. Fraction, Density, and Average Size of Dislocation Loops Under Different Irradiation Directions

**Figure 1:**
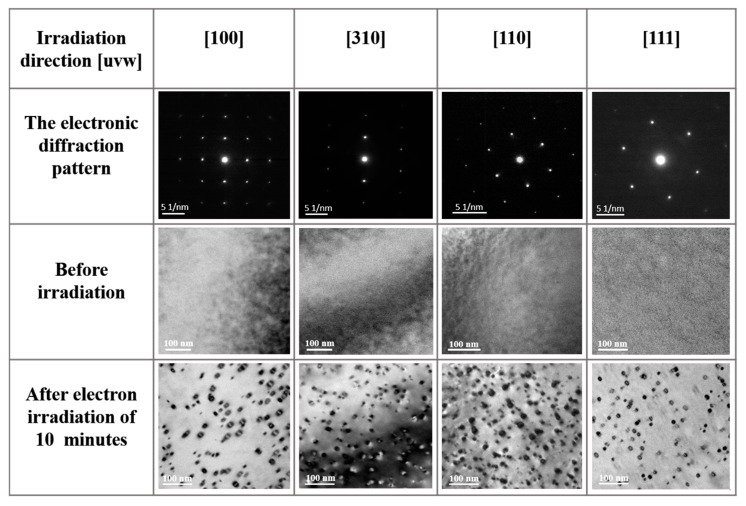
Selected-area electron diffraction (SAED) patterns and TEM bright-field images of the sample acquired along the [100], [310], [110], and [111] crystallographic orientations, obtained before and after 10 min of electron irradiation.

Prior to electron irradiation, the TEM bright-field images show that the annealed pure Al samples contain no dislocations or other defects. After irradiation with 200 keV electrons for 10 min along the [100], [310], [110], and [111] crystallographic orientations, high densities of small dislocation loops appeared within the irradiated regions, as revealed in Figure 1.

**Figure 2:**
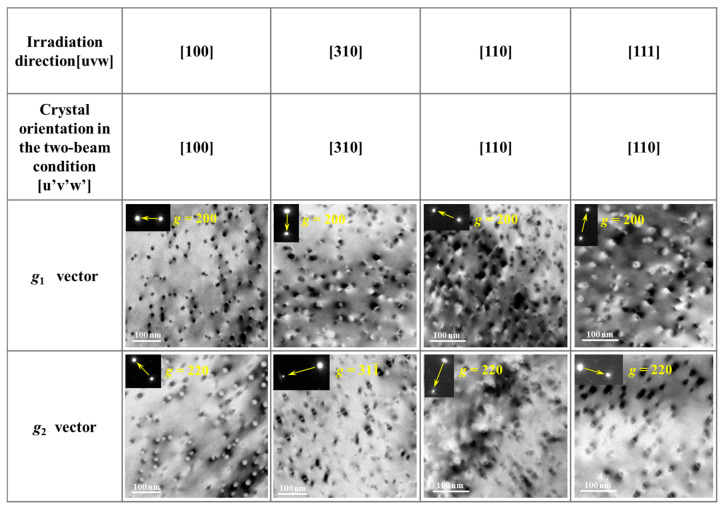
Bright-field TEM images under two-beam conditions showing dislocation loops after 10 min of electron irradiation along the [100], [310], [110], and [111] orientations. Different diffraction ***g*** vectors were used as indicated in each image.

As an example, under [100] irradiation, after 10 min of electron irradiation, the dislocation loops exhibited an average diameter d^[100]^ of 14.4 nm and a number density ρ^[100]^ of 5.1 × 10^21^/m^3^, as shown in Figure 2. Because the first-order diffraction vectors in the SAED pattern for the [111] are all ***g*** = {220}, which is unfavorable for detailed characterization of the dislocation loops, the [110] zone axis was selected instead. Under this condition, two diffraction vectors, ***g*** = {200} and ***g*** = {220}, were employed to characterize the dislocation loops generated during electron irradiation along the [111] direction, as shown in Figure 2. The fractions of <111> and <110> dislocation loops were quantified based on their extinction behavior under the two ***g*** vectors. The extinction rules for both types of loops under the selected diffraction conditions are summarized in Table 1.

**Figure 3:**
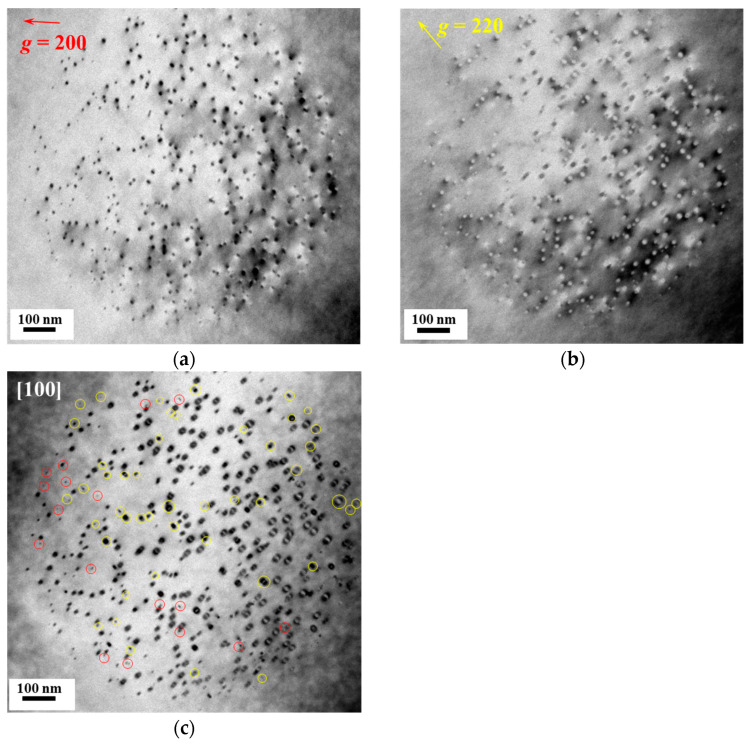
Bright-field TEM images after 10 min of electron irradiation along the [100] direction: (**a**) two-beam condition with ***g*** = {200}, (**b**) two-beam condition with ***g*** = {220}, and (**c**) [100] crystallographic orientation.

After 10 min of electron irradiation along the [100] direction, bright-field TEM images were obtained under three imaging conditions, as shown in Figure 3: the two-beam condition with (a) ***g*** = {200}, (b) ***g*** = {220}, and (c) along the [100] crystallographic orientation. Statistical analysis indicates that under the ***g*** = {200} condition, a total of 412 dislocation loops were counted, among which two-thirds of the <110> loops and all <111> loops were visible. Under the ***g*** = {220} condition, 385 dislocation loops were observed, with five-sixths of the <110> loops and half of the <111> loops visible. Using Equation (1), the numbers of <110> and <111> dislocation loops (N_<110>_ and N_<111>_) were calculated to be 358 and 173, respectively. The fraction of <111> loops among the total dislocation loops, $P_{<111>}^{L\;}$, is 33%, while that of <110> loops, $P_{<110>}^{L\;}$, is 67%. Based on the observed area and the sample thickness (~100 nm), the densities of <111> and <110> dislocation loops were determined to be ρ_<111>_ = 1.7 × 10^21^/m^3^ and ρ_<110>_ = 3.4 × 10^21^/m^3^, respectively.

(1) $$\left\{\;\begin{array}{c} \frac{2}{3}N_{<110>}+N_{<111>}=412\\ \frac{5}{6}N_{<110>}+\frac{1}{2}\;N_{<111>}=385 \end{array}\right.$$

The dislocation loops that were extinct under specific two-beam conditions were identified by comparing bright-field TEM images shown in Figure 3. The loops that became extinct under the ***g*** = {200} condition (Figure 3a) were marked with red circles in the [100] crystallographic orientation image (Figure 3c), while those extinct under the ***g*** = {220} condition (Figure 3b) are indicated by yellow circles in Figure 3c. The extinction behavior of dislocation loops under different irradiation directions and two-beam conditions is summarized in Table 1. After electron irradiation along the [100] direction, all loops extinct under the ***g*** = {200} condition were of the <110> type, whereas all <111> loops remained visible. Under the ***g*** = {220} condition, one-third of the extinct loops were <110> type, while two-thirds were <111> type. The average loop sizes generated during irradiation along the [100] direction were calculated to be 22.5 nm for <111> loops and 10.4 nm for <110> loops.

Using the approach described above, the fractions, average sizes, and densities of <111> and <110> dislocation loops were determined after electron irradiation along the [310], [110], and [111] directions, and the results are summarized in Table 2. Significant differences in loop densities, ρ^[uvw]^, were observed for different irradiation directions, where [uvw] denotes the irradiation direction. The highest loop density was observed along the [111] direction, while the lowest occurred along [100]. The loop densities for the four irradiation directions decreased in the order: ρ^[111]^ (1.2 × 10^22^/m^3^) > ρ^[110]^ (1.0 × 10^22^/m^3^) > ρ^[310]^ (7.8 × 10^21^/m^3^) > ρ^[100]^ (5.1 × 10^21^/m^3^). Similarly, the average loop sizes, d^[uvw]^, also varied with irradiation direction, following the trend: d^[100]^ (14.4 nm) > d^[310]^ (12.5 nm) > d^[110]^ (12.4 nm) > d^[111]^ (11.3 nm).

Significant differences were observed in the fractions of <111> and <110> dislocation loops ($P_{<\mathrm{hkl}>}^{L\;[\mathrm{uvw}]}$) generated under different irradiation directions, as summarized in Table 2. $P_{<\mathrm{hkl}>}^{L\;[\mathrm{uvw}]}$ represents the percentage of <hkl>-type loops among the total number of loops for the [uvw] irradiation direction. For the [100] and [310] irradiation directions, $P_{<111>}^{L\;}$ was lower than $P_{<110>}^{L\;}$. Along the [100] direction, $P_{<111>}^{L\;}$ was 33% and $P_{<110>}^{L\;}$ was 67%, while along the [310], $P_{<111>}^{L\;}$ was 43% and $P_{<110>}^{L\;}$ was 57%. In contrast, for the [110] and [111] directions, $P_{<111>}^{L\;}$ was higher than $P_{<110>}^{L\;}$. Along the [110] direction, $P_{<111>}^{L\;}$ was 90% and $P_{<110>}^{L\;}$ was 10%, whereas along [111], $P_{<111>}^{L\;}$ was 78% and $P_{<110>}^{L\;}$ was 22%. The relative magnitude of $P_{<111>}^{L\;}$ among the four irradiation directions decreased in the order [110] > [111] > [310] > [100], whereas that of $P_{<110>}^{L\;}$ followed [100] > [310] > [111] > [110].

The densities of <111> and <110> dislocation loops generated along different irradiation directions were calculated using $\rho_{<\mathrm{hkl}>}^{\left[\mathrm{uvw}\right]}$= ρ^[uvw]^$P_{<\mathrm{hkl}>}^{L\;[\mathrm{uvw}]}$ as summarized in Table 2. The ρ_<111>_ values for the different irradiation directions decreased in the order: [111] > [110] > [310] > [100], with the maximum $\rho_{<111>}^{\left[111\right]}$ = 9.4 × 10^21^/m^3^ and the minimum $\rho_{<111>}^{\left[100\right]}$ = 1.7 × 10^21^/m^3^, giving a factor of 5.5 between the largest and smallest values. Similarly, the ρ_<110>_ values followed the order [310] > [100] > [111] > [110], with the maximum $\rho_{<110>}^{\left[310\right]}$ = 4.5 × 10^21^/m^3^ and the minimum $\rho_{<110>}^{\left[110\right]}$ = 1.0 × 10^21^/m^3^, corresponding to a factor of 4.5 between the largest and smallest densities.

Comparison of <111> and <110> dislocation loops generated along the same irradiation direction revealed that d_<111>_ was consistently larger than d_<110>_. For the [100], [310], and [110] irradiation directions, d_<111>_ was approximately twice that of d_<110>_, whereas for the [111] direction, the average sizes of the two loop types were comparable. Both d_<111>_ and d_<110>_ reached their maximum values under [100] irradiation, 22.5 nm and 10.4 nm, respectively, which were significantly larger than the corresponding values in the other directions. The relative magnitudes of d_<111>_ for the four irradiation directions decreased in the order [100] > [310] > [110] > [111], with the maximum approximately twice the minimum. For d_<110>_, the order was [100] > [111] > [310] > [110], with the maximum being 1.6 times the minimum.

### 3.2. IAD in Dislocation Loops Generated by Irradiation in Different Directions

The analysis above indicated that, although the dislocation loop densities decreased in the order ρ^[111]^ > ρ^[110]^ > ρ^[310]^ > ρ^[100]^, the average loop sizes followed d^[100]^ > d^[310]^ > d^[110]^ > d^[111]^. This suggested that irradiation directions that produced smaller average loop sizes tended to correspond to higher loop densities. It is well established that dislocation loops generated in aluminum under 200 keV electron irradiation at room temperature are of interstitial type [29,30]. Therefore, the IAD within <111> and <110> loops was calculated for different irradiation directions to provide further insight into the effect of irradiation direction on dislocation loop evolution.

**Figure 4:**
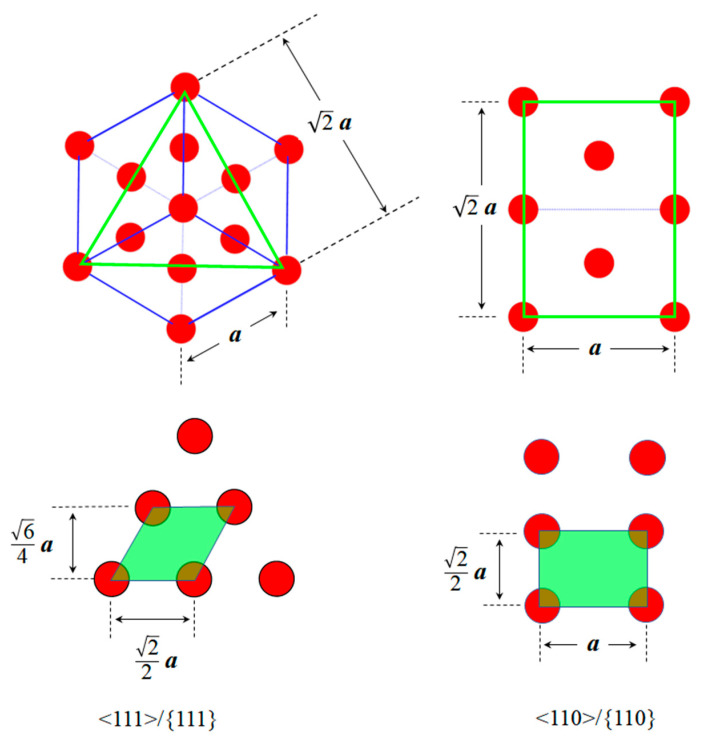
Atomic planar densities of the {111} and {110} planes in an FCC crystal.

In FCC crystal structures, <111> dislocation loops have a habit plane belonging to the {111} planes, whereas <110> loops have a habit plane belonging to the {110} planes. As shown in Figure 4, the area occupied by a single atom on the (111) plane (green parallelogram) is $\frac{\sqrt{3}}{4}a^{2}$, resulting in an atomic planar density of $\frac{4\sqrt{3}}{3a^{2}}$ for the (111) plane. Similarly, the area occupied by a single atom on the (110) plane (green rectangle) is $\frac{\sqrt{2}}{2}a^{2}$, giving an atomic planar density of $\frac{\sqrt{2}}{a^{2}}$ for the (110) plane, where ***a*** is the lattice parameter. At room temperature, the lattice parameter of Al is 4.05 × 10^−10^ m [39]. Accordingly, the atomic planar densities of the {111} and {110} plane families were calculated to be 1.41 × 10^19^ atoms/m^2^ and 8.62 × 10^18^ atoms/m^2^, respectively.

The IAD within <hkl> dislocation loops along different irradiation directions, denoted as $C_{<\mathrm{hkl}>}^{\left[\mathrm{uvw}\right]}$, was calculated using the following equation:

(2) $${C_{<\mathrm{hkl}>}^{\;[\mathrm{uvw}]}=\;S_{<\mathrm{hkl}>}^{\;[\mathrm{uvw}]}\rho }_{<\mathrm{hkl}>}^{\left[\mathrm{uvw}\right]}A\left(\mathrm{hkl}\right)$$

In Equation (2), $C_{<\mathrm{hkl}>}^{\;\left[\mathrm{uvw}\right]}$ represents the IAD within <*hkl*>-type dislocation loops along the [*uvw*] irradiation direction, with units of atoms/m^3^. Physically, it corresponded to the number of SIAs contained in <*hkl*> loops per unit volume generated during electron irradiation. $S_{<\mathrm{hkl}>}^{\;\left[\mathrm{uvw}\right]}$ denotes the average area of <*hkl*> dislocation loops along the [*uvw*] direction, with units of m^2^. $\rho_{<\mathrm{hkl}>}^{\left[\mathrm{uvw}\right]}$ is the number density of <*hkl*> loops along the [*uvw*] direction, in units of m^−3^. A_(hkl)_ is the atomic planar density of the habit plane of <*hkl*>-type dislocation loops, with units of atoms/m^2^.

(3) $$S_{<\mathrm{hkl}>}^{\;[\mathrm{uvw}]}=\;\pi {\left(\frac{d_{<\mathrm{hkl}>}^{\;[\mathrm{uvw}]}}{2}\right)}^{2}$$

In Equation (3), $d_{<\mathrm{hkl}>}^{\;[\mathrm{uvw}]}$ denotes the average size of <*hkl*>-type dislocation loops along the [*uvw*] irradiation direction, with units of m. π is the mathematical constant, taken as approximately 3.14. By substituting Equation (3) into Equation (2), Equation (4) was obtained:

(4) $$C_{<\mathrm{hkl}>}^{\;[\mathrm{uvw}]}=\pi {\left(\frac{d_{<\mathrm{hkl}>}^{\;[\mathrm{uvw}]}}{2}\right)}^{2}\rho_{<\mathrm{hkl}>}^{\left[\mathrm{uvw}\right]}A_{<\mathrm{hkl}>}$$

By substituting the results from Table 2 into Equation (4), the IAD values in <111> and <110> dislocation loops, $C_{<\mathrm{hkl}>}^{\;[\mathrm{uvw}]}$, were calculated for the four irradiation directions, where [uvw] denotes the electron beam direction, and <hkl> represents the dislocation loop type. For example, $C_{<111>}^{\;[110]}$ corresponds to the IAD within <111> loops generated under [110] irradiation. The total IAD contained in dislocation loops with the two kinds of Burgers vectors under different irradiation directions, $C_{i}^{\;[\mathrm{uvw}]}$, is defined as $C_{i}^{\;[\mathrm{uvw}]}\;={\;C}_{<111>}^{\;[\mathrm{uvw}]}+C_{<110>}^{\;[\mathrm{uvw}]}$; for instance, $C_{i}^{\;[110]}$ represents the sum of $C_{<111>}^{\;[110]}$ and $C_{<110>}^{\;[110]}$, i.e., the total IAD within dislocation loops after irradiation along [110]. The atomic fractions of <111> and <110> loops along [uvw], denoted as $P_{<111>}^{A\;[\mathrm{uvw}]}$ and $P_{<110>}^{A\;[\mathrm{uvw}]}$, were also determined. The detailed results are summarized in Table 3, which shows IAD values in <111> and <110> dislocation loops under the four irradiation directions.

For all irradiation directions, both C_<111>_ and $P_{<111>}^{A}$ were significantly higher than C_<110>_ and $P_{<110>}^{A}$. Under [110] irradiation, the IAD $C_{<111>}^{\;[110]}$ and $C_{<110>}^{\;[110]}$ were 1.7 × 10^25^ atoms/m^3^ and 3.0 × 10^23^ atoms/m^3^, respectively, representing the largest difference among the four irradiation directions; $C_{<111>}^{\;[110]}$ was approximately 56 times higher than $C_{<110>}^{\;[110]}$. The corresponding atomic fractions, $P_{<111>}^{A\;[110]}$ and $P_{<110>}^{A\;[110]}$, were 98.3% and 1.7%, indicating that <111> loops contributed the vast majority of SIAs under [110] irradiation.

**Figure 5:**
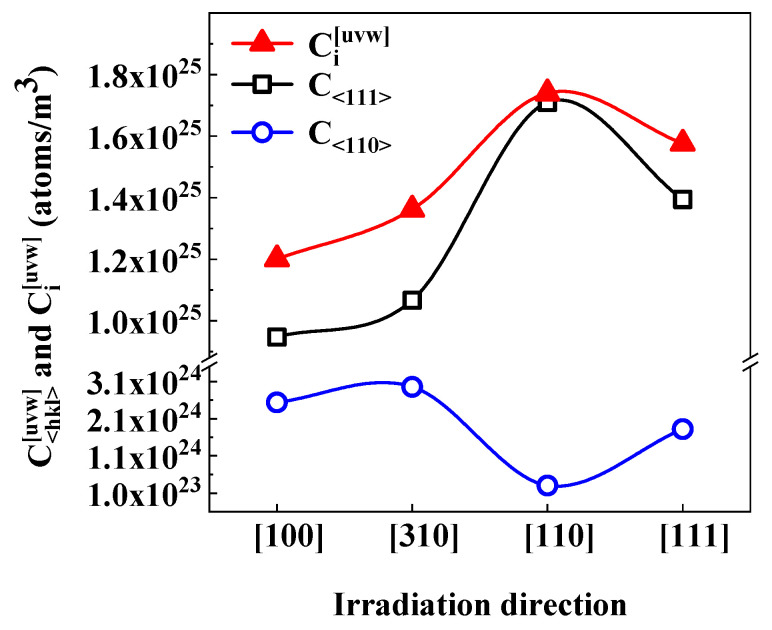
$C_{<\mathrm{hkl}>}^{\;[\mathrm{uvw}]}$ and $C_{i}^{\;[\mathrm{uvw}]}$ within dislocation loops under electron irradiation along four crystallographic orientations.

Across all irradiation directions, the IAD of C_<111>_ decreased in the order [110] > [111] > [310] > [100], whereas C<110> followed [100] > [310] > [111] > [110]. The total IAD in the two types of loops, $C_{i}^{\;[\mathrm{uvw}]}$, showed the maximum and minimum values of $C_{i}^{\;[110]}$ = 1.7 × 10^25^ atoms/m^3^ and $C_{i}^{\;[100]}$ = 1.2 × 10^25^ atoms/m^3^, respectively. The results for $C_{<\mathrm{hkl}>}^{\;[\mathrm{uvw}]}$ and $C_{i}^{\;[\mathrm{uvw}]}$ are summarized in Figure 5. As shown, the trends in $C_{<\mathrm{hkl}>}^{\;[\mathrm{uvw}]}$ and $C_{i}^{\;[\mathrm{uvw}]}$ were consistent, which was attributed to the dominant contribution of <111> loops to IAD, reflected by the high values of $P_{<111>}^{A\;\left[\mathrm{uvw}\right]}$.

## 4. Discussion

### 4.1. Effect of Direction-Dependent E_d_ on Evolution of Dislocation Loops

After 10 min of electron irradiation along different crystallographic orientations, the total IAD in dislocation loops decreased in the order $C_{i}^{\;[110]}$ (1.7 × 10^25^ atoms/m^3^) > $C_{i}^{\;[111]}$ (1.6 × 10^25^ atoms/m^3^) > $C_{i}^{\;[310]}$ (1.4 × 10^25^ atoms/m^3^) > $\;C_{i}^{\;[100]}$ (1.2 × 10^25^ atoms/m^3^). This variation is primarily attributed to the direction dependence of the E_d_. E_d_ is defined as the minimum energy required for an incident particle to produce a stable Frenkel pair (a self-interstitial atom and a vacancy) during irradiation-induced damage. Under room temperature electron irradiation, the anisotropy of E_d_ among different crystallographic orientations has a significant impact on the concentration of SIAs.

Previous studies have shown that the concentration of displaced atoms generated under an incident electron flux during irradiation is given by [16]

*N_d_* = *n_i_tφσ_p_ν*(*E_p_*) (5)

where *N_d_* is the concentration of displaced atoms, with units of atoms/m^3^, *n_i_* is the atomic density of the specimen, with units of atoms/m^3^, *t* is the irradiation time (s), *φ* is the incident electron flux, with units of electrons/(s∙m^2^), *σ_p_* is the primary total scattering cross-section of the incident electrons, and *ν*(*E_p_*) is the displacement damage function. According to the NRT model [40], for 200 keV electron irradiation of pure Al, the displacement damage function is *ν*(*E_p_*) = 1 during irradiation.

When the quantum effects of electrons are taken into account, the primary total scattering cross-section *σ_p_* of the incident electrons is given by [16]

(6) $$\sigma_{p}=\frac{\pi Z^{2}e^{4}(1-\beta^{2})}{\beta^{2}m_{0}^{2}c^{4}}\left\{\frac{E_{p,\max}}{E_{d}}-1-\beta^{2}\ln\frac{E_{p,\max}}{E_{d}}+\pi \alpha \beta \left[2{\left(\frac{E_{p}}{E_{p,\max}}\right)}^{\frac{1}{2}}-2-\ln\frac{E_{p,\max}}{E_{d}}\right]\right\}$$

*Z* is the atomic number of the target material, *e* is the elementary charge, and *β* is the relative velocity of the electrons, defined as *β = v*/*c*, where *c* is the speed of light and *v* is the velocity of the incident electrons. *m*_0_ is the electron rest mass. *E_p,max_* denotes the maximum transferable energy from an electron to an atom (in eV), and *E_d_* is the displacement threshold energy of the material (in eV). The parameter *α* is defined as *Z*/137. *E_p_* represents the energy transferred from an incident electron to a target atom (in eV). The corresponding expressions are as follows:

(7) $$E_{p}\;=\;E_{p,\max}{(\sin\frac{\theta }{2})}^{2},0\;\leq \;\theta \;\leq \;\pi$$

*θ* is the angle between the motion direction of the displaced atom and the incident electron beam. According to the calculations reported by Yin et al. on the relationship between TEM accelerating voltage and the energy transferred to target atoms, a 200 keV electron can transfer a maximum energy of E_p,max_ = 19.5 eV to an Al atom [30]. For 200 keV electrons, β = 0.7, π = 3.14, and α = 0.09. Substituting these values into Equation (6) yields the following:

(8) $$\sigma_{p}\;=\;\frac{\pi Z^{2}e^{4}(1-\beta^{2})}{\beta^{2}m_{0}^{2}c^{4}}\left(\frac{19.5}{E_{d}}-0.69\ln\frac{19.5}{E_{d}}\;-1.4\;+\;0.4\sin\frac{\theta }{2}\;\right)$$

The concentration of displaced atoms, *N_d_*, does not necessarily equal the concentration of SIAs, *C_d_*, because a displaced atom may recombine with other vacancies and could potentially be displaced multiple times during irradiation. According to previous studies, the atomic displacement efficiency, *ξ*, is close to 1 for electrons and light particles, indicating a low recombination rate between SIAs and vacancies under these irradiation conditions [15] (pp. 152–154). Therefore, under electron irradiation, the concentration of SIAs can be approximated as being equal to that of displaced atoms:

*C_d_* ≈ *N_d_* (9)

In this study, the irradiation temperature, as well as the area and thickness of the irradiated region, were comparable among the samples. Moreover, the atomic density of the specimens *n_i_*, the irradiation time *t*, the electron flux *φ*, the atomic number of the target *Z*, the elementary charge *e*, the relative velocity of electrons *β*, and the electron mass m_0_ were all constants. Therefore, two constants, *k*_1_ and *k*_2_, can be introduced:

(10) $$k_{1}\;=\;n_{i}t\varphi \frac{\pi Z^{2}e^{4}(1-\beta^{2})}{\beta^{2}m_{0}^{2}c^{4}},k2\;=\;-1.4\;+\;0.4\sin\frac{\theta }{2}$$

*k*_1_ > 0 and −1.4 *≤ k*_2_ ≤ 1. The concentration of SIAs, *C_d_*, can then be expressed as

(11) $$C_{d}=k_{1}\left(\frac{19.5}{E_{d}}-0.69\ln\frac{19.5}{E_{d}}+{\;k}_{2}\right)$$

Equation (11) illustrates the relationship between E_d_ and C_d_ under electron irradiation, indicating that a lower E_d_ results in a higher C_d_. Higher SIA concentrations provide favorable conditions for the nucleation and growth of dislocation loops, thereby enhancing the IAD within dislocation loops $C_{i}^{\;[\mathrm{uvw}]}$. Based on the obtained irradiation results, $C_{i}^{\;[110]}$ > $C_{i}^{\;[111]}$ > $C_{i}^{\;[310]}$ > $C_{i}^{\;[100]}$, it can be inferred that the corresponding E_d_ values for the four irradiation directions follow the order $E_{d}^{\;[110]}\;<\;E_{d}^{\;[111]}<{\;E}_{d}^{\;[310]}<E_{d}^{\;[100]}$.

Tanaka et al. [35] measured the E_d_ of pure Al along different crystallographic orientations at 120 °C using the dislocation loop growth method. Their results showed that among the [100], [110], and [111] directions, [100] exhibited the highest E_d_ of 17 eV, which is consistent with the inference in this study. The high E_d_ along the [100] implies that Al atoms in the crystal lattice are less likely to be displaced by electrons, reducing the probability of atomic displacement and resulting in a lower SIA concentration, C_d_, in agreement with the lowest $C_{i}^{\;[100]}$ observed in the present work. Nevertheless, the minimum E_d_ reported by Tanaka et al. was along [111] (14 eV), rather than [110] (16 eV), which is inconsistent with our inference. For FCC metals, the [110] is generally considered to have the lowest E_d_, as reported for Cu [37,41] and Ni [42,43]. Hohenstein et al. [44] noted that the relatively high electron energy used during irradiation in Tanaka et al.’s experiments leads to more complex electron–lattice interactions, introducing significant uncertainty in the derived threshold surface. They further noted that if the irradiation electron energy were adjusted to 150–180 keV, the direction of minimum E_d_ in Al would be expected to shift to [110].

In addition, irradiation temperature can significantly affect the measured E_d_ [41]. Studies on the temperature dependence of E_d_ in Cu indicate that E_d_ increases with temperature for all orientations within a certain range, with the increase being more pronounced for [100] and [110] than for [111] [45]. Therefore, we speculate that the experimental temperature of 120 °C in Tanaka et al.’s study likely caused a sharp decrease in E_d_ along [111], resulting in the observed minimum E_d_ in that orientation.

It should be noted that the relative magnitudes of E_d_ along different characteristic directions in pure Al at room temperature have yet to be determined by precise measurements using the dislocation loop growth method [36], resistivity method [46], or reduced-voltage method [30,37].

### 4.2. Effect of 1D Migration of <110> Dislocation Loops

Another noteworthy experimental observation is that, under the [110] irradiation condition, both the density of <110> dislocation loops ($\rho_{<110>}^{\left[110\right]}$) and the IAD within these loops ($C_{<110>\;}^{\;[110]}$) are anomalously low, as shown in Figure 5. The IAD contained in loops is affected by the loop type, density, and size, all of which are governed by the nucleation and growth behavior of loops during irradiation. In general, a higher loop nucleation rate tends to produce a larger loop density, whereas a higher loop growth rate leads to larger loop sizes.

The nucleation and growth of dislocation loops are influenced by multiple factors, including the production rate of SIAs during irradiation, the recombination rate between SIAs and vacancies [32,33,34], the sink bias of dislocation loops with different Burgers vectors, the diffusion of SIAs, and, in particular, the long-range one-dimensional migration. The 1D migration of dislocation loops has been observed in various metals, such as Al, Fe, and Cu [29,47,48,49,50,51,52]. During 1D migration, loops undergo rapid motion along their Burgers vector direction. Li et al. [29] reported that <110> loops in pure Al can experience fast 1D migration at room temperature under electron irradiation, with a maximum migration distance up to 1500 nm. Such long-range, high-velocity 1D migration of loops significantly enhances the diffusion and transport of SIAs.

In pure aluminum, the stacking fault energy is high, and <111> loops correspond to faulted Frank loops. Such loops do not exhibit 1D migration along their Burgers vector and can only evolve through climb. In contrast, <110> dislocation loops are capable of 1D migration along their Burgers vectors [16,50,52,53]. This 1D migration of <110> loops can strongly affect the observable loop density. In this study, among the four irradiation orientations examined, the influence of 1D migration on loop density is most pronounced under [110] irradiation. This is because the ±[110] directions, which are perpendicular to the specimen surface, are available for loop migration during irradiation along [110]. Considering that the long-range 1D migration distance of dislocation loops in Al can greatly exceed the thickness of a TEM foil, <110> loops migrating along ±[110] can readily escape to the specimen surface.

Therefore, the remarkably low IAD within <110> loops, $C_{<110>\;}^{\;[110]}$, obtained after [110] irradiation, is very likely attributable to the long-range 1D migration-assisted escape of <110> dislocation loops to the foil surface.

## 5. Conclusions

The study employed a 200 keV TEM electron beam to conduct in situ irradiation experiments on pure aluminum along four different crystallographic orientations. All irradiations were performed at room temperature for a duration of 10 min. The main conclusions are as follows:

(1) The total density of dislocation loops formed under the four irradiation directions decreases in the order [111] > [110] > [310] > [100]. Among them, the loop densities produced under [111] and [110] irradiation are similar, both approximately twice those under [100] irradiation. The average loop sizes are relatively close, with the following order from largest to smallest: [100] > [310] > [110] > [111].
(2) A comparison between the two Burgers vector types, <111> and <110> dislocation loops, shows that for all irradiation directions, the <110> loops exhibit smaller diameters than the <111> loops. Under [100] and [310] irradiation, the density of <111> loops is lower than that of <110> loops, whereas the opposite trend is observed under [110] and [111] irradiation. In particular, under [110] irradiation, the density of <111> loops is nine times that of <110> loops. This behavior is attributed to the fact that <111> loops in aluminum are difficult to migrate, whereas <110> loops can undergo long-range one-dimensional migration, resulting in a lower retained density.
(3) The calculated interstitial atom density contained within the loops shows that, for different irradiation directions, the interstitial density decreases in the order [110] > [111] > [310] > [100]. Based on these results, the threshold displacement energy, E_d_, of pure aluminum under electron irradiation at room temperature is inferred to follow the crystallographic orientation dependence: $E_{d}^{\;[110]}<E_{d}^{\;[111]}<E_{d}^{\;[310]}<{\;E}_{d}^{\;[100]}$.

## Figures and Tables

**Table 1:** Extinction behavior of dislocation loops after electron irradiation.

Irradiation Direction [uvw]		[100]		[310]		[110]		[111]	
Two-Beam Conditions Along Crystallographic Direction [u’v’w’]		[100]		[310]		[110]		[110]	
	*g*	(200)	(220)	(200)	($31\overset{-}{1}$)	(200)	(220)	(200)	(220)
$\overset{⃑}{b}$									
1/2 [110]		√	√	√	√	√	√	√	√
1/2 [101]		√	√	√	√	√	√	√	√
1/2 [011]		×	√	×	×	×	√	×	√
$1/2\;[1\overset{-}{1}$0]		√	×	√	√	√	×	√	×
$1/2\;[\overset{-}{1}$01]		√	√	√	√	√	√	√	√
$1/2\;[0\overset{-}{1}$1]		×	√	×	√	×	√	×	√
1/3 [111]		√	√	√	√	√	√	√	√
$1/3\;[1\overset{-}{1}$1]		√	×	√	×	√	×	√	×
$1/3\;[11\overset{-}{1}$]		√	√	√	√	√	√	√	√
$1/3[\overset{-}{1}$11]		√	×	√	√	√	×	√	×

√: dislocation loop visible; ×: dislocation loop extinct (not visible).

**Table 2:** Size, density, and fraction of <111> and <110> loops under different irradiation directions.

	Irradiation Direction [uvw]			
	[100]	[310]	[110]	[111]
Total dislocation loop density ρ^[uvw]^ (/m^3^)	5.1 × 10^21^	7.8 × 10^21^	1.0 × 10^22^	1.2 × 10^22^
Average dislocation loops size d^[uvw]^ (nm)	14.4	12.5	12.4	11.3
Fraction of <111> loops $P_{<111>}^{L\;}$ (%)	33	43	90	78
Fraction of <110> loops $P_{<110>}^{L\;}$ (%)	67	57	10	22
Density of <111> loops ρ_<111>_ (/m^3^)	1.7 × 10^21^	3.4 × 10^21^	9.0 × 10^21^	9.4 × 10^21^
Density of <111> loops ρ_<110>_ (/m^3^)	3.4 × 10^21^	4.5 × 10^21^	1.0 × 10^21^	2.6 × 10^21^
Average size of <111> loops d_<111>_ (nm)	22.5	17.5	13.1	11.6
Average size of <110> loops d_<110>_ (nm)	10.4	8.7	6.7	10.1

**Table 3:** IAD in <111> and <110> dislocation loops under four irradiation directions.

Irradiation Direction [uvw]	Dislocation Loop Type <hkl>	C_<111>_ and C_<110>_ (atoms/m^3^)	$C_{i}^{\;[\mathrm{uvw}]}$ (atoms/m^3^)	$P_{<111>}^{A\;}$$\;\mathrm{and}\;P_{<110>}^{A\;}$ (%)
[100]	<111>	9.4 × 10^24^	1.2 × 10^25^	79.0
	<110>	2.5 × 10^24^		21.0
[310]	<111>	1.1 × 10^25^	1.4 × 10^25^	83.4
	<110>	2.3 × 10^24^		16.6
[110]	<111>	1.7 × 10^25^	1.7 × 10^25^	98.3
	<110>	3.0 × 10^23^		1.7
[111]	<111>	1.4 × 10^25^	1.6 × 10^25^	88.4
	<110>	1.8 × 10^24^		11.6

## Data Availability

The original contributions presented in this study are included in the article. Further inquiries can be directed to the corresponding author.
